# Bilateral Mandibular Paramolars

**DOI:** 10.5005/jp-journals-10005-1231

**Published:** 2014-04-26

**Authors:** Kanika Singh Dhull, Rachita Singh Dhull, Swagatika Panda, Sonu Acharya, Shweta Yadav, Gatha Mohanty

**Affiliations:** Reader, Department of Pedodontics and Preventive Dentistry, Kalinga Institute of Dental Sciences, KIIT University, Bhubaneswar Odisha, India; Fellow, Department of Pediatric Nephrology, Detroit Medical Center Michigan, USA; Senior Lecturer, Department of Oral and Maxillofacial Pathology, Institute of Dental Sciences, SOA University, Bhubaneswar, Odisha, India; Reader, Department of Pedodontics and Preventive Dentistry Institute of Dental Sciences, SOA University, Bhubaneswar Odisha, India; Reader, Department of Prosthodontics, PDM Dental College Bahadurgarh, Haryana, India; Lecturer, Department of Pedodontics and Preventive Dentistry Institute of Dental Sciences, SOA University, Bhubaneswar Odisha, India

**Keywords:** Supernumerary tooth, Paramolars, Hyperdontia

## Abstract

Supernumerary tooth is a developmental anomaly and has been argued to arise from multiple etiologies. These teeth may remain embedded in the alveolar bone or can erupt into the oral cavity. They can cause a variety of complications in the develo­ping dentition. Supernumerary teeth can present in various forms and in any region of the mandible or maxilla, but have a predisposition for the anterior maxilla. Here is the presentation of a case of unusual location of supernumerary teeth located in between mandibular first and second molar region bilaterally.

**How to cite this article: **Dhull KS, Dhull RS, Panda S, Acharya S, Yadav S, Mohanty G. Bilateral Mandibular Paramolars. Int J Clin Pediatr Dent 2014;7(1):40-42.

## INTRODUCTION

Supernumerary teeth, or hyperdontia, can be defined as the teeth that exceed the normal dental complement, regardless of their location and morphology. Supernumerary teeth may occur singly, multiply, unilaterally or bilaterally and in one or both jaws.^1^ Paramolars or distomolars are the supernumerary teeth in the molar region. A paramolar is a supernumerary molar, usually rudimentary, situated buccally or lingually/ palatally to one of the molars or in the interproximal space buccal to the second and third molar.^2^

The prevalence of supernumerary teeth in permanent dentition varies from 0.5 to 3.8%, in com parison to 0.3 to 0.6% in primary dentition. Supernumerary teeth appear with a higher frequency in men than in women, with a 2:1 ratio.^3^ Supernumerary teeth can occur as singles, multiples, unilaterally or bilaterally and in the maxilla, the mandible or both. Supernumerary teeth are estimated to occur in the maxilla 8.2 to 10 times more frequently than the mandible, and most commonly affect the premaxilla.^2^ Cases involving one or two supernumerary teeth most commonly affect the anterior maxilla, followed by the mandibular premolar region. Paramolars and distomolars occur less frequently in the mandible than in the maxilla.^4^

This is the presentation of a case of unusual location of supernumerary teeth located in-between mandibular first and second molar region bilaterally.

## CASE REPORT

**Fig. 1:**
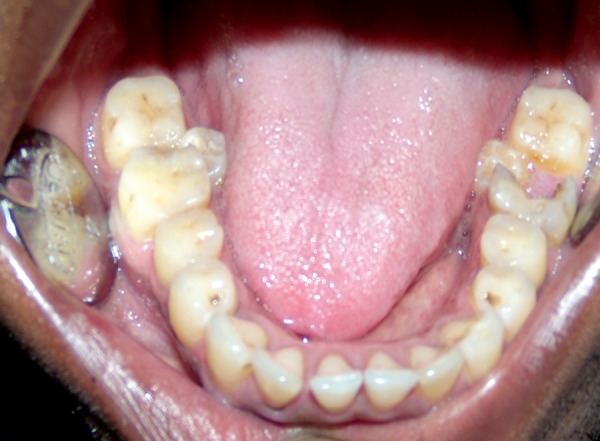
Clinical intraoral picture showing paramolars mesial and lingual to mandibular second molar bilaterally

**Fig. 2:**
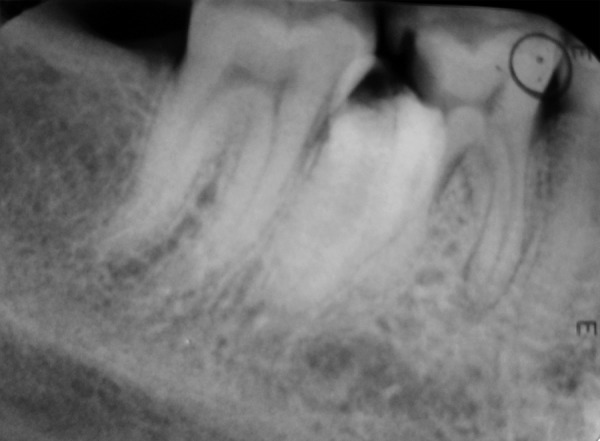
Intraoral radiograph showing paramolar (right side)

**Fig. 3:**
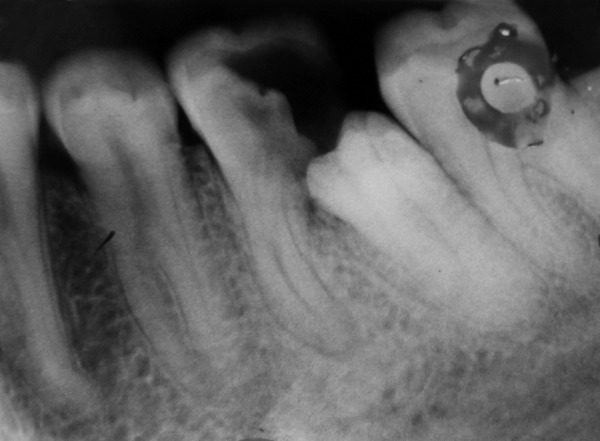
Intraoral radiograph showing paramolar (left side)

An 18 years old male patient reported to the private dental clinic, Bhubaneswar, Odisha, India, with the chief complaint of pain in lower left back region. The patient's medical and dental history was noncontributory. Intraoral exami­nation revealed the presence of moderate gingivitis, grossly carious lower left first molar, proximal deep dental caries in lower right first molar and supernumerary teeth mesial and lingual to mandibular second molar bilaterally (Fig. 1). The patient was not aware of their existence and the occur­rence of supernumerary teeth in other family members. The supernumerary tooth resembled mandibular second molar morphologically. Intraoral periapical radiographs were taken to see any radiological findings (Figs 2 and 3). The patient did not give consent for the extraction of paramolars as he did not have any problem with the presence of extra teeth. The lower left first molar was extracted and root canal treat­ment was done on lower right first molar.

## DISCUSSION

Supernumerary teeth are a common clinical and radiographic finding and may produce occlusal and dental problems. An unerupted supernumerary tooth may be found by chance during radiographic examination, with no effect on adjacent teeth. It is essential not only to enumerate but also to identify the supernumerary teeth present clinically and radiographi-cally before a definitive diagnosis and treatment plan can be formulated. Classification of supernumerary teeth may be on the basis of position or form.^5^ Positional variations include mesiodens, paramolars, distomolars and parapre-molars. Variations in form consist of conical types, tuber-culate types, supplemental teeth and odontomes. Super­numerary teeth may, therefore, vary from a simple odontome, through a conical or tuberculate tooth to a supplemental tooth which closely resembles a normal tooth. Supernumerary teeth are less common in the deciduous dentition with a reported incidence of 0.3 to 1.7% of the population.^6^ Possible exp­lanations for the less frequent reporting of deciduous super­numerary teeth include less detection by parents, as the spacing frequently encountered in the deciduous dentition may be utilized to allow the supernumerary tooth or teeth to erupt with reasonable alignment.

The etiology is unknown, although a number of theories have been proposed: atavism, tooth germ dichotomy, hyper-activity of the dental lamina and genetic factors comprising a dominant autosomal trait characterized by low penetrance.^7^

Supernumerary teeth may occur in isolation or as part of a syndrome. The most common syndromes that show a significant incidence of multiple supernumerary teeth are cleft lip and palate, cleidocranial dysostosis and Gardner's syndrome. Less common developmental disorders are Fabry Anderson's syndrome, Chondroectodermal dysplasia (Ellis-Van Creveld syndrome), Ehlers-Danlos syndrome, inconti-nentia pigmenti and Trico-Rhino-Phalangeal syndrome.^8^

Supernumerary teeth may erupt normally, remain impacted, appear inverted or assume an abnormal path of eruption.^9^ Crowding may be evident due to an increased number of erupted teeth. Failure of eruption of adjacent permanent teeth is the most frequent occurrence and occurs in 30 to 60% of cases.^5^ The supernumerary or adjacent teeth may be displaced and ectopic eruption of either is not uncommon. Supernumerary teeth may also cause diastemata, root resorption of adjacent teeth, malformation of adjacent teeth, such as dilaceration, and loss of vitality of adjacent teeth.^10^

There are different treatment modialities described in literature for patients with multiple hyperdontia not associated to complex syndromes. Treatment is partly dependent upon the position and clinical manifestations of the super­numerary tooth. Thus, an early diagnosis is very important in order to decide among extraction, extraction followed by orthodontic treatment, or simply monitorization or control of the supernumerary teeth, with a view to minimizing the risk of complications secondary to the presence of these teeth. Surgical management in turn ranges from removal of the supernumerary teeth to removal of the latter followed by orthodontic treatment aiming to ensure correct occlusion. In the more complex cases, the possible existence of multiple impactions of supernumerary teeth gives rise to destructuring of the dental arch, with numerous malpositioned teeth. These situations require close cooperation among professionals to define combined surgical orthodontic management.^7^

## CONCLUSION

Supernumeraries can cause a variety of complications. The clinician should recognize signs suggesting the presence of supernumerary teeth, particularly aberrations sin the erup­tive pattern, and perform the relevant investigations. On diagnosis, each case should be managed appropriately in order to minimize complications to the developing dentition.
